# An Automated Phylogenetic Tree-Based Small Subunit rRNA Taxonomy and Alignment Pipeline (STAP)

**DOI:** 10.1371/journal.pone.0002566

**Published:** 2008-07-02

**Authors:** Dongying Wu, Amber Hartman, Naomi Ward, Jonathan A. Eisen

**Affiliations:** 1 UC Davis Genome Center, University of California Davis, Davis, California, United States of America; 2 Section of Evolution and Ecology, College of Biological Sciences, University of California Davis, Davis, California, United States of America; 3 Department of Medical Microbiology and Immunology, School of Medicine, University of California Davis, Davis, California, United States of America; 4 Department of Molecular Biology, University of Wyoming, Laramie, Wyoming, United States of America; 5 Center of Marine Biotechnology, Baltimore, Maryland, United States of America; 6 The Johns Hopkins University, Department of Biology, Baltimore, Maryland, United States of America; Ecole Normale Supérieure de Lyon, France

## Abstract

Comparative analysis of small-subunit ribosomal RNA (ss-rRNA) gene sequences forms the basis for much of what we know about the phylogenetic diversity of both cultured and uncultured microorganisms. As sequencing costs continue to decline and throughput increases, sequences of ss-rRNA genes are being obtained at an ever-increasing rate. This increasing flow of data has opened many new windows into microbial diversity and evolution, and at the same time has created significant methodological challenges. Those processes which commonly require time-consuming human intervention, such as the preparation of multiple sequence alignments, simply cannot keep up with the flood of incoming data. Fully automated methods of analysis are needed. Notably, existing automated methods avoid one or more steps that, though computationally costly or difficult, we consider to be important. In particular, we regard both the building of multiple sequence alignments and the performance of high quality phylogenetic analysis to be necessary. We describe here our fully-automated ss-rRNA taxonomy and alignment pipeline (STAP). It generates both high-quality multiple sequence alignments and phylogenetic trees, and thus can be used for multiple purposes including phylogenetically-based taxonomic assignments and analysis of species diversity in environmental samples. The pipeline combines publicly-available packages (PHYML, BLASTN and CLUSTALW) with our automatic alignment, masking, and tree-parsing programs. Most importantly, this automated process yields results comparable to those achievable by manual analysis, yet offers speed and capacity that are unattainable by manual efforts.

## Introduction

### ss-RNA gene sequence analysis as a tool for microbial systematics and ecology

Phylogenetic analysis of rRNA gene sequences (particularly ss-rRNA, i.e., the small subunit rRNA) has led to important advances in microbiology, such as the discovery of a third branch on the tree of life (the archaea) [1] and the realization that the microbes that can be grown in pure culture represent but a small fraction, in terms of both phylogenetic types and total numbers of cells of the microbes, found in nature [2]. The power of ss-rRNA for phylogenetic analysis can be attributed to many factors, including its presence in all cellular organisms, its favorable patterns of sequence conservation that enable study of both recent and ancient evolutionary events, and the ease with which this gene can be cloned and sequenced from new organisms [3]. The sequencing of ss-rRNA genes from new species is greatly facilitated by the presence of highly conserved regions at several positions along the gene [4]. The conservation of these regions allows one to design and use broadly targeted oligonucleotide primers that work on a wide diversity of species for both sequencing and amplification by the polymerase chain reaction (PCR). In fact, it is now standard procedure to sequence the ss-rRNA gene when a new microbe has been isolated [5], [6].

The ss-rRNA gene has become a key target for environmental microbiology studies largely because through the use of broadly targeted primers, one can use PCR to amplify in a single reaction the ss-rRNA genes from a wide diversity of organisms present in an environmental sample [7], [8]. The amplified products can then be characterized in multiple ways such as through restriction digestion [9], [10], denaturing gradient gel electrophoresis [11], hybridization to arrays [12], or sequencing. As sequencing continues to decrease in cost and difficulty, we believe it will become the preferred option and thus we focus on sequence analysis here.

Once DNA sequences of environmental ss-rRNA genes are in hand, multiple types of analyses can be used to characterize the organisms and communities from which they were obtained. For example phylogenetic analysis of the sequences can reveal what types of microbial organisms are present in a sample. In addition, very closely related ss-rRNA sequences can be grouped together into *phylotypes* or *operational taxonomic units* (OTUs), groupings which often serve as a provisional surrogate for “species.” From these groupings one can then estimate the total number of species (i.e., the species richness) and their *relative abundance* [13].

### Limitations of ss-RNA gene sequence analysis

As powerful as it is as a tool for phylogenetic and environmental analysis, it is important to point out that analyses based on ss-rRNA are not without limitations. For example, there is significant variation between species in the number of copies of the ss-rRNA gene present in the genome. This makes it challenging to use the number of sequences one obtains for particular phylotypes in an environment to estimate the relative abundance of those phylotypes [14]. Another limitation lies in the use of PCR amplification. Though very broadly targeted PCR primers are frequently referred to as “universal” in that they are supposed to amplify all members of a major taxonomic group (e.g., all bacteria, or all archaea), even the best designed ones are not as universal as the moniker implies [15]. All primer sets tend to preferentially amplify genes from some evolutionary group preferentially over others making both quantification and even presence/absence information sometimes not representative of the community. A third limitation of ss-rRNA in general (for both cultured and uncultured organisms) is that phylogenetic trees of ss-rRNA genes do not always accurately reflect the complete history of an organism [16], [17]. This inaccuracy can be due to many factors including artifacts (e.g., bad alignments), biased data sets (e.g., convergent evolution or highly variable rates of evolution between taxa), or lateral gene transfer (which could cause even a perfectly inferred rRNA tree to be different from the general phylogeny of the organism). Thus it is always desirable to include protein phylogenetic markers in addition to ss-rRNAs in any microbial diversity study.

In terms of sequence-based characterization of communities, metagenomic methods, which involve the random sequencing of all the DNA or RNA from environmental samples [18], [19] have been seen as a potential replacement for rRNA-based studies. Metagenomics is indeed very powerful in that it circumvents some of the limitations of PCR methods and, in the process, generates sequence data for many other genes from organisms present in a community. Thus metagenomics can not only characterize the types of organisms present, but can also be used to predict the diverse activities which can be carried out by the community [20]. Metagenomics has even led to the discovery of novel lineages of organisms completely missed by rRNA PCR [21].

Although some view metagenomic analysis as a replacement for PCR based rRNA studies, we see it as a complementary approach. The power of metagenomics comes from its broad sampling of all the DNA present in a community. However, this approach works best for characterizing abundant organisms. rRNA-PCR, because it targets only a single gene, allows one to better sample the less abundant organisms in a community. In addition, the same technological advances that underlie the increased use of metagenomic methods have also made it possible to obtain ss-rRNA sequences more cheaply and in greater numbers than ever before [22]. We think it is important to note that metagenomic sequencing does produce ss-rRNA sequences which can be analyzed in much the same way as PCR-generated ss-rRNAs. This serves as an important cross check for both approaches. Whether the source of the sequences is rRNA-PCR or metagenomic sequencing, one of the great advantages of focusing analyses on ss-rRNA sequences studies is the ever-expanding database of ss-rRNA sequences from cultured organisms and environmental samples [23]–[25].

### Goals of ss-RNA gene sequence analysis

Overall, the number of ss-rRNA sequences being determined with PCR and metagenomics from environmental samples is increasing exponentially. Analysis of this rapidly accumulating wealth of ss-rRNA sequences has raised a significant challenge – how to balance the desire for high quality methods with the need for automation to keep up with the sequence onslaught. To understand what methods are needed for ss-rRNA sequence analysis, we believe it is important to focus on the some of the key results that are the goal of many studies. These include: (1) delineation of OTUs present in a set of sequences, (2) assignment of sequences (or a representative of each OTU) to taxonomic groups, (3) generation and use of phylogenetic trees of all or some sequences, frequently including previously determined sequences as well.

With a small number of sequences, there is a simple path to generating relatively high quality outputs for each of the above three desired results. First, one generates a multiple sequence alignment including both the new sequences and those from a ribosomal RNA collection. From the alignment one can identify OTUs (desired result #1) and build phylogenetic trees (desired result #3). These trees might be used to search for phylogenetic groups found only in certain environments or to perform community to community comparisons using methods such as UniFrac [26]. In addition, from the trees one can then assign sequences to taxonomic groups (desired result #2) by looking at the taxonomy of nearby sequences in the tree. Though each researcher or group might have a preferred approach to each of these steps, the general outline (multiple sequence alignment first, then phylogenetic and sequence analysis second) is highly similar for those who have desired high quality analyses of a small number of sequences.

A variety of software tools are available for researchers to carry out multiple sequence alignments and phylogenetic analyses of rRNA genes. Perhaps the most widely used is the software package ARB [27], which allows users to carry out a wide diversity of rRNA based analyses and also to share compatible resources between labs. Despite its power, there are several challenges to using ARB for massive collections of ss-RNA sequences including that alignments need to be manually created within ARB and that taxonomic assignments require visual inspection of trees and manual input from users. For example, two workers analyzing a dataset of 11,831 sequences, spent ∼5 months manually aligning and annotating their ss-RNA dataset in the ARB software package (Elies Bik, personal communication)[28].

### The need for automation, and limitations of existing automated methods

The curation of alignments and the building of phylogenetic trees by manual methods is labor intensive and time-consuming, and, importantly, the results are often subjective and depend heavily on the skill and expertise of the user. Notably, many microbial ecologists using these tools are not formally trained in phylogenetics or comparative sequence analysis. Furthermore, as more and more sequences have and will become available, manual analysis is increasingly unfeasible.

Over the last 5–10 years multiple automated tools have been developed to aid in the analysis of ss-rRNA sequences. In fact, methods are available to produce each of the desired results outlined above. However, there are limitations to most of these tools in that they tend to avoid using multiple sequence alignments or true phylogenetic analyses as part of their approach. Most likely this is done in order to obtain speed or because automation of multiple alignment and phylogeny was deemed unfeasible. However, this we believe can compromise the value of the results.

For example, the delineation of OTUs has also been automated via tools that do not make use of alignments or phylogenetic trees (e.g., Greengenes). This is usually done by carrying out pairwise comparisons of sequences and then clustering of sequences that have better than some cutoff threshold of similarity with each other). This approach can be powerful (and reasonably efficient) but it too has limitations. In particular, since multiple sequence alignments are not used, one cannot carry out standard phylogenetic analyses. In addition, without multiple sequence alignments one might end up comparing and contrasting different regions of a sequence depending on what it is paired with.

The limitations of avoiding multiple sequence alignments and phylogenetic analysis are readily apparent in tools to classify sequences. For example, the Ribosomal Database Project's Classifier program [29] focuses on composition characteristics of each sequence (e.g., oligonucleotide frequency) and assigns taxonomy based upon clustering genes by their composition. Though this is fast and completely automatable, it can be misled in cases where distantly related sequences have converged on similar composition, something known to be a major problem in ss-rRNA sequences [30]. Other taxonomy assignment systems focus primarily on the similarity of sequences. The simplest of these is to use BLASTN to search a sequence database (e.g., Genbank) and to then use information about the top match to assign some sort of taxonomy information to new sequences. Such similarity-based approaches are analogous to using top blast matches to predict the functions of genes and have similar limitations. Though fast, such approaches are not ideal because the most similar sequence may not in fact be the most closely related sequence due to the vagaries of evolution such as unequal rates of change in different lineages or convergent evolution [31]–[35].

Despite the clear advantages of using multiple sequence alignments and phylogenetic analyses for many aspects of ss-rRNA analyses, there are only a few examples of attempts to generate these outputs in a highly or completely automated manner. The most comprehensive tool we are aware of is the BIBI software package [36], which takes new sequences, identifies similar sequences in a database using BLASTN and then generates a new multiple sequence alignment and then produces phylogenetic trees from the alignment. Users can then view the trees to make taxonomic assignments based upon phylogenetic position of query sequences relative to known ones. Though BIBI is quantum leap more advanced than most similarity based available classification tools it does have some limitations. For example, the generation of new alignments for each sequence is both computational costly, and does not take advantage of available curated alignments that make use of ss-RNA secondary structure to guide the primary sequence alignment. Perhaps most importantly however is that the tool is not fully automated. In addition, it does not generate multiple sequence alignments for all sequences in a dataset which would be necessary for doing many analyses.

Automated methods for analyzing rRNA sequences are also available at the web sites for multiple rRNA centric databases, such as Greengenes and the Ribosomal Database Project (RDPII). Though these and other web sites offer diverse powerful tools, they do have some limitations. For example, not all provide multiple sequence alignments as output and few use phylogenetic approaches for taxonomy assignments or other analyses. More importantly, all provide only web-based interfaces and their integrated software, (e.g., alignment and taxonomy assignment), cannot be locally installed by the user. Therefore, the user cannot take advantage of the speed and computing power of parallel processing such as is available on linux clusters, or locally alter and potentially tailor these programs to their individual computing needs (Table 1).

**Table 1:** Comparison of STAP's computational abilities relative to existing commonly-used ss-RNA analysis tools.

	STAP	ARB	Greengenes	RDP
Installed where?	Locally	Locally	Web only	Web only
User interface	Command line	GUI	Web portal	Web portal
Parallel processing	YES	NO	NO	NO
Manual curation for taxonomy assignment	NO	YES	NO	NO
Manual curation for alignment	NO	YES	NO*	NO
Open source	YES**	NO	NO	NO
Processing speed	Fast	Slow	Medium	Medium

It is important to note, that STAP is the only software that runs on the command line and can take advantage of parallel processing on linux clusters and, further, is more amenable to downstream code manipulation.

* Note: Greengenes alignment output is compatible with upload into ARB and downstream manual alignment.

** The STAP program itself is open source, the programs it depends on are freely available but not open source.

Given the limited automated tools that are available for researchers have had to choose between two non-ideal options: manually generating and/or curating alignments (an expensive and slow process which can handle only a limited number of sequences) or using the non-phylogenetic and non-alignment based methods that can be automated more readily.

We describe here the development of a fully-automated, high-throughput method that meets many of the key requirements of ss-rRNA sequence analysis. First, this method generates high quality multiple sequence alignments that can be used for phylogenetic reconstructions as well as for diversity measures such as the identification of OTUs. Secondly, the method generates a phylogenetic tree for each query sequence and assigns that sequence to a taxonomic group based upon its position in the tree relative to other known sequences. The alignments and phylogenetic tree outputs of this program can be used for input into a variety of other software tools such as DOTUR (for identifying OTUs) and UNIFRAC (for phylogenetic based community comparisons)[26], [37]. We refer to this method as STAP: a **S**mall Subunit rRNA **T**axonomy and **A**lignment **P**ipeline.

A key advantage of STAP is that it is the only fully automated method available that can be locally installed by the user and is independent of a web-based interface. One benefit of this is that it allows the use of parallel processing (when implemented on linux clusters, STAP has the capacity to process large numbers of ss-rRNA sequences). Another advantage of STAP is that it is open-source and amenable to improvement or alternate applications and can be adapted into workflow software. In this paper we describe the STAP software, its testing, and provide examples of some of the ways it can be used in ss-rRNA analyses.

## Methods

### Building the database

The database currently used by STAP is populated by data retrieved from two ss-rRNA sequence databases: bacterial and archaeal sequences from Greengenes [24], and eukaryotic sequences from RDP II [23]. In preparing the database, our goal was to create a compact set of well-annotated sequences representing every major phylogenetic group in all three domains. Some multiple sequence alignments and taxonomies derived from that data set are also included in the database, as detailed below.

#### Preparation of BLASTN-ready sequences

Bacterial and archaeal ss-rRNA sequences, including some environmental sequences assigned to these domains, were extracted as a multiple sequence alignment from the Greengenes database [24]. Likewise, eukaryotic 18S rRNA sequences and related environmental sequences were extracted as a multiple sequence alignment from the RDP II database [23].

For more efficient downstream processing, steps were taken to eliminate the sequence redundancy existing in the imported data. All sequences were searched against each other using BLASTN. A Lek clustering algorithm [38] was then used to cluster sequences that showed greater than 99% identity over at least 80% of their length—criteria which typically grouped sequences at the species level. In each cluster, those sequences with more detailed taxonomic annotations were selected to represent the cluster, and other sequences were discarded. These collections of selected BLASTN-ready sequences are referred to as the *complete data sets*, one for bacterial/archaeal sequences and one for eukaryote sequences. A BioPerl [39] sequence index was built for each data set for sequence and alignment retrieval.

#### Alignments and taxonomies

Alignments of the bacterial and archaeal ss-rRNA sequences were downloaded from the Greengenes database [24], while alignments of all the eukaryotic ss-rRNAs were retrieved from the RDP II database [23]. Taxonomy information was prepared in XML format for the sequences in each set. Taxonomic identification for bacteria and archaea is based on the P. Hugenholtz taxonomy from the Greengenes database [24], that for eukaryotes is from the RDP II database [23]. An XML data structure and XML::DOM module were used for storing and retrieving taxonomy information.

#### Representative subsets

To provide distant sequences for tree balancing and rooting, a *representative subset* that included sequences from each of the major phylogenetic groups was identified for each of the complete data sets. The archaeal/bacterial subset contained 20 archaeal and 195 bacterial ss-rRNA sequences; the eukaryote subset contained 136 sequences. A BioPerl [39] sequence index was built for each subset for sequence and alignment retrieval.

#### The three-domain subset

A separate *three-domain subset* including sequences from all three domains of life was prepared solely for use in assigning query sequences to a domain. The alignments used for this purpose were retrieved from the European rRNA database [25] which aligns bacterial and archaeal rRNA sequences with eukaryote sequences. Twenty archaeal, 186 bacterial, and 134 eukaryotic sequences previously selected for the representative subsets were included. The alignments were manually curated and trimmed prior to use with STAP and are available as part of the STAP package.

### Processing ss-rRNA query sequences

Using STAP, individual query sequences are analyzed by a three-step process, with each step employing both sequence alignment and phylogenetic analysis. The first step assigns the sequence to a domain, the second makes a provisional, approximate assignment to a taxonomic group, and the third refines the analysis to assign the sequence to a lower-level taxonomic group.

**Figure 1:**
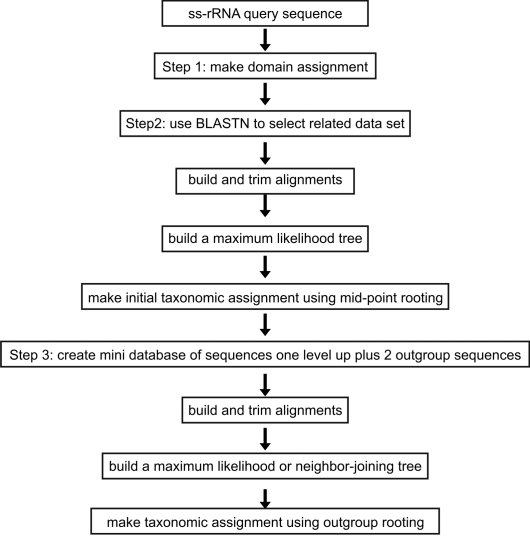
A flow chart of the STAP pipeline.

STAP automates three principle tasks that are required by those three steps: the selection of pertinent homologous sequences for use in the analysis; building, masking, and trimming multiple sequence alignments; building and analyzing phylogenetic trees. See Figure 1 for a flow chart of the STAP pipeline.

### Step 1: Assigning sequences to a domain

In the first step, the query sequence is compared to the three-domain subset described above in order to assign the sequence to a domain. This step can be omitted if the domain affiliation is known. The domain assignment made at this step determines which database will be used for subsequent analyses, an important consideration since alignments were built separately for the eukaryotic sequence database and the bacterial and archaeal sequence database.

**Figure 2:**
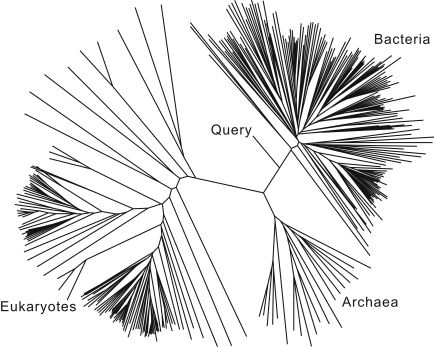
Domain assignment. In Step 1, STAP assigns a domain to each query sequence based on its position in a maximum likelihood tree of representative ss-rRNA sequences. Because the tree illustrated here is not rooted, domain assignment would not be accurate and reliable (sequence similarity based methods cannot make an accurate assignment in this case either). However the figure illustrates an important role of the tree-based domain assignment step, namely automatic identification of deep-branching environmental ss-rRNAs.

A query sequence is aligned to the three-domain subset using the CLUSTALW profile alignment method [40]. Next, a maximum likelihood tree is built from the alignment by PHYML (substitution model: HKY; Transition/transversion ratio∶4.0; Proportion of invariable sites: program estimated; Number of substitution rate categories: 1; Gamma distribution parameter∶1.0; Starting tree: BIONJ distance-based tree; No starting tree optimization) [41]. A tree parser perl script then identifies the nodes that separate the domains, and also the node that specifies the position of the query sequence on the tree (Figure 2). Based on these relative positions, a domain is assigned to the query sequence.

### Step 2: Assigning sequences to subgroups within a domain

#### Selecting a related data set

In theory, one could locate a query sequence within a domain by performing a phylogenetic analysis of *all* sequences in that domain. However, the number of sequences within each domain is too large to use, even for high-throughput phylogenetic analysis. Therefore, we developed the following method to first identify a subset of related sequences which could then be used for more detailed phylogenetic analysis.

First, BLASTN is used to search the query sequence against the complete sequence data set for that domain. Matches are ranked by E-value and the top 50 are selected for further analysis. Since it is known that the closest relatives of a sequence are not always included in the top BLASTN matches, we also include 10 lower scoring hits by selecting every 100^th^ record in the BLASTN rankings (i.e., the 150^th^ hit, the 250^th^, the 350^th^, etc.) [31]. To further balance the analysis and ensure that possible close relatives are not missed, we also search the query sequence against the corresponding representative subset and retrieve the top 10 matches. Thus, a *related data set* containing 70 sequences is prepared for use in subsequent analyses.

#### Alignment, masking, and trimming

The alignments for the 70 sequences in the related data set are extracted from the STAP database, and the query sequence is aligned to them using the CLUSTALW profile alignment algorithm [40] as described above for domain assignment. By adapting the profile alignment algorithm, the alignments from the STAP database remain intact, while gaps are inserted and nucleotides are trimmed for the query sequence according to the profile defined by the previous alignments from the databases. Thus the accuracy and quality of the alignment generated at this step depends heavily on the quality of the Bacterial/Archaeal ss-rRNA alignments from the Greengenes project or the Eukaryotic ss-rRNA alignments from the RDPII project.

Phylogenetic analysis using multiple sequence alignments rests on the assumption that the residues (nucleotides or amino acids) at the same position in every sequence in the alignment are homologous. Thus, columns in the alignment for which “positional homology” cannot be robustly determined must be excluded from subsequent analyses. This process of evaluating homology and eliminating questionable columns, known as masking, typically requires time-consuming, skillful, human intervention. We designed an automated masking method for ss-rRNA alignments, thus eliminating this bottleneck in high-throughput processing.

First, an alignment score is calculated for each aligned column by a method similar to that used in the CLUSTALX package [42]. Specifically, an R-dimensional sequence space representing all the possible nucleotide character states is defined. Then for each aligned column, the nucleotide populating that column in each of the aligned sequences is assigned a score in each of the R dimensions (Sr) according to the IUB matrix [42]. The consensus “nucleotide” for each column (X) also has R dimensions, with the score for each dimension (*Xr*) calculated as the average of the scores for that column in that dimension (average of *Sr*). Thus the score of the consensus nucleotide is a mathematical expression describing the average “nucleotide” in that column for that alignment.

**Figure 3:**
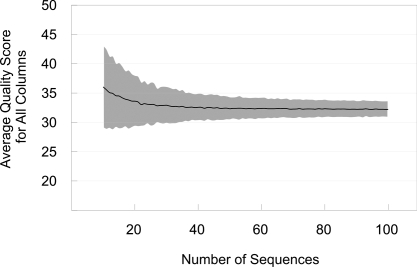
Determination of the quality score cutoff for automated alignment trimming. The average quality score for all columns for alignments of randomly-generated sequences is plotted against the number of sequences in the alignment (see Methods). Standard deviations are indicated by gray shading.

Additional calculations are made which provide a measure of the sequence diversity in the alignment. The distance between nucleotide *i* in the column and the consensus nucleotide X is defined as *Di* and is calculated by:
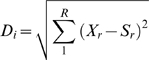



The alignment quality score *Q* is calculated for each column using the following equation where *D_average_* is the average of the nucleotide distances *Di*, m is the total number of sequences in the alignment, and *n* is the number of nucleotides in that column:
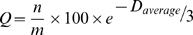



Highly diverse columns embedded in highly conserved regions are thought not to be the result of poor alignments. To prevent such potentially informative columns from being masked and trimmed, the alignment score of column *i* is adjusted to its neighbors in a 7-nucleotide sliding window according to the equation:
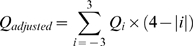



Automated trimming requires an alignment quality cutoff score to be specified, i.e., a way to identify when an alignment is significantly better than random. We used the following strategy to determine the alignment quality score that would result from random alignments of similar nucleotide composition. For bacterial and archaeal ss-rRNAs, 195 representative ss-rRNA sequences were searched against the STAP database using BLASTN. Alignments were retrieved for each of the representative sequences as described above. For each alignment, columns with more than 80% gaps were eliminated and the average nucleotide composition of the remaining columns from all the alignments was calculated. Since the alignments for STAP analysis typically contained 70 sequences, 2000 random columns were generated for an alignment of 70 sequences of that average nucleotide composition and each column was assigned a quality score. The calculated average scores and standard deviations indicate what scores could be expected for purely random alignments. The results for alignments containing varying numbers of sequences are shown in Figure 3. A similar procedure carried out for eukaryote ss-rRNA yielded comparable results (data not shown). Based on the data shown in Figure 3, a cutoff of 31 was selected as a “better than random” quality score. Only columns with a quality score of 31 or higher are retained when masking bacterial, archaeal, and eukaryotic ss-rRNA alignments for automated trimming.

#### Phylogenetic analysis

A phylogenetic tree is constructed for the masked and trimmed alignments provided by the previous step. By default, STAP generates maximum likelihood trees using the PHYML [41] program (substitution model: HKY; Transition/transversion ratio∶4.0; Proportion of invariable sites: program estimated; Number of substitution rate categories: 1; Gamma distribution parameter∶1.0; Starting tree: BIONJ distance-based tree; Optimize the topology, the branch lengths and rate parameters of the starting neighbor-join tree optimization).

STAP processes the tree file using a PERL module to mid-point root the tree. During processing, all nodes in the tree are captured by the module and the relationships among them are stored in a two-dimensional matrix. The module walks from the query sequence to the root, recording the nodes as well as the taxonomic identification of the leaves en route in a tab-delimited text file.

STAP then attempts to assign the query sequence to a taxonomic group based on its closest neighbors in the tree. STAP will look at the first six neighbors searching for a sequence that has been assigned to a taxonomic group in our ss-rRNA database.

### Step 3: Refining the alignments and taxonomic assignments

#### Selecting a data set

Starting with the initial taxonomic assignment generated in Step 2, STAP identifies the taxonomic group one level above that assignment. (The user has the option to specify using the taxonomic group two or more levels up, instead.) STAP builds a *mini database* containing all sequences from this group and then applies the procedures used in Step 2 to create a subset of sequences from this mini database. In this instance, however, the subset of sequences selected for further analysis is made up of 62 sequences: the top 50 matches, plus ten lower-scoring hits selected at 100 record intervals, plus two sequences selected from other taxonomic groups to serve as outgroups for tree rooting.

#### Alignment, masking, trimming, and analysis

These procedures are carried out as in Step 2 with one difference: the tree is rooted using the outgroups identified based on the preliminary taxonomic assignment, as described above.

### STAP ss-rRNA aligner

The STAP ss-rRNA aligner takes one ss-rRNA sequence as the input and outputs a gapped sequence that aligned to the user's chosen alignment database. The user has to chose one database, and the STAP aligner first searches the query sequences against the user's chosen database (bacteria/archaea or eukaryotes) by BLASTN. Alignments of the top 20 hits are extracted from the corresponding alignment dataset. A mask is generated to document positions of the all-gapped columns in the extracted alignment and the all-gapped columns are subsequently trimmed to produce an alignment profile. The query sequence is aligned on top of the profile by the CLUSTALW profile alignment algorithm. Only the aligned query sequence is kept, and gaps are inserted back to the aligned query sequences according to the all-gap mask. All the sequences aligned to the same database are concatenated into one file; the user can use a perl script provided in the STAP package to remove all-gapped columns to get the final alignment.

### Comparison of BLASTN and STAP taxonomic assignments

To check the accuracy of taxonomic assignments made by STAP, we compared STAP assignments with those derived from the top BLASTN match—a commonly used high-throughput method. To this end, we selected the bacterial sequences in the complete STAP database which had more than six levels of taxonomic annotation. An “all vs. all” BLASTN search was performed for the selected sequences. A Lek clustering algorithm [38] was then used to cluster sequences that showed greater than 95% identity over at least 80% of their length. This left 823 sequences.

The selected sequences were then each processed against all ss-rRNA sequences by two methods: by using STAP with the default maximum likelihood tree option selected, and by using BLASTN. Since STAP will search as many as six close neighbors in the tree seeking a sequence with a high-quality taxonomic assignment, the parsing by BLASTN was likewise directed to examine up to six neighbor sequences to identify one with a quality taxonomic annotation. The taxonomy assignments obtained by the two methods were compared to the original Hugenholtz annotation [24]. Trees built by the pipeline also served as references to validate the taxonomic assignments.

## Results and Discussion

### The automated tasks

The STAP pipeline automates three principle tasks: the selection of pertinent homologous sequences for analysis; the building, masking, and trimming of multiple sequence alignments; and the building and analyzing of phylogenetic trees.

#### Automated selection of homologous sequences

One of the challenges facing any phylogenetic study based on ss-rRNA sequences is the enormous and ever-growing number of available sequences. Though it is theoretically possible to build a tree using *all* of those sequences, it is simply computationally too costly to do so for high-throughput processing. This is particularly true for STAP since it is designed to analyze each new ss-rRNA sequence individually. If an environmental sample contained 10,000 rRNA sequences, building trees with homologs for each of the 10,000 would be excessively costly. We note that this issue arises for virtually any method of phylogenetic analysis when many homologs are available, not just for automated methods.

Therefore, we sought to design a method that would select a group of homologous sequences that would be sufficient for accurate taxonomic identification of each new ss-rRNA sequence, yet be computationally lean. We estimated that for analyses of environmental samples containing tens of thousands of ss-rRNAs each, we would be limited to using only 50–100 sequences in the building of each tree. Given this constraint, procedures were required that could reduce the redundancy in the database and that could, for each new ss-rRNA sequence, select a subset of 50–100 sequences capable of yielding an accurate taxonomic identification.

To eliminate redundancy in the database, we designed a simple clustering method to identify sets of very closely related sequences (see Methods). Only the best annotated sequence from each set was used for construction of the STAP “complete” data sets. Only 42% of the bacterial and archaeal ss-rRNAs in the July 2006 release of the Greengenes Project database met this criterion for importation into the STAP data sets.

To select a suitable subset of homologous sequences for the phylogenetic analysis, we included not only the best BLASTN matches found in the complete STAP database, but also selected a set of lower scoring sequences. In addition, we selected the best matches from a search against the representative data set, thus ensuring that several different major lineages would be included in the analysis.

#### Automated generation of multiple sequence alignments

After homologous sequences have been selected, building a sequence alignment is the next critical step. Ideally, ss-rRNA sequence alignments would use the conserved secondary structure to guide the primary sequence alignment. For sequences which lack close relatives in the database, the use of a structurally-based alignment method is even more important. Unfortunately, such structurally-based alignment algorithms are too expensive computationally for this type of high-throughput analysis. Therefore, we did the next best thing, i.e., we used the pre-existing, curated alignments of large numbers of ss-rRNA sequences in the Greengenes database. To align query sequences to these pre-existing alignments, we used the CLUSTALW profile alignment algorithm. This algorithm has been shown to work quite well when the database contains at least a few moderately close relatives of the query sequence.

Phylogenetic analysis methods treat each alignment column separately and assume that any given column has a common evolutionary history for all sequences in the alignment. Therefore, any ambiguous regions in the alignment should be excluded from subsequent analyses. This process of identifying and removing questionable columns, referred to as masking and trimming, typically requires tedious manual intervention. STAP includes an automated masking and trimming method which is based not only on the degree of conservation of individual columns, but on the conservation of neighboring columns, as well (see Methods). The masked alignments produced by STAP's automated process agreed well with those produced by our manual curation (data not shown).

#### Automated phylogenetic tree construction and parsing

The tree-building program that STAP adapts is PHYML [41], which implements a maximum-likelihood algorithm.

Accurate taxonomic assignments require that the phylogenetic trees be rooted. STAP uses the midpoint method of rooting when making the initial taxonomic assignment to a subgroup (Step 2, above), and uses outgroups for rooting the final tree (Step 3, above).

For analysis of the resultant trees, we developed a PERL program to evaluate each tree automatically without compromising the benefits of manual analysis. The program scans through a tree to capture all the nodes, and then walks from the query sequence to the root. Along the way it records the nodes and taxonomic information for the leaves encountered in a tab-delimited text file.

### Speed and Throughput

#### Balancing speed and accuracy

STAP incorporates a three-step process designed to balance processing speed with phylogenetic accuracy. Step 1 assigns a sequence to a domain of life, thus specifying which database will be used for further analyses. Step 2 makes an initial approximate taxonomic identification of the query sequence within that domain. Step 3 does a fine-scale phylogenetic analysis within the taxonomic group identified in Step 2. Since the assignments made in Step 2 are sometimes inaccurate at the low taxonomic level (e.g. genus level), by default the analysis in Step 3 starts with all the sequences from the taxonomic group at the next higher level. Notably, the tree built and analyzed in Step 3 is rooted using outgroups identified in Step 2.

#### Processing time

A key feature of STAP is its speed. When running on a Fedora Core 5 linux machine with a 3.73 GHz Dual Core IntelXeon processor and 2GB RAM, it takes STAP an average of 1 minute and 35.4 seconds to assign taxonomy to a query sequence by the maximum likelihood tree-building method if the domain information is provided by the user. Thus, a single machine can process up to 500 sequences in a few hours. For samples containing thousands of sequences, a linux cluster is recommended. A small cluster of 20 nodes can process 1000 environmental ss-rRNA sequences in less than 2 hours. On clusters with more than a hundred nodes, which are becoming more common, STAP can process thousands of sequences in a matter of minutes.

### Reliability

#### STAP versus BLASTN

Many researchers have turned to BLASTN searches as a means of rapidly classifying the flood of new ss-rRNA sequences, despite the large body of literature showing that BLASTN searches are not a robust way to identify the closest relatives in a sequence database [31], [32]. Phylogenetic methods, such as those employed by STAP, are generally considered far superior for that purpose as these methods can take the variation in evolutionary rates and other vagaries of evolution into account. In addition, it is important to point out that STAP also produces high-quality multiple sequence alignments which can be used for a variety of other analyses including studies of species richness and relative abundances.

**Figure 4:**
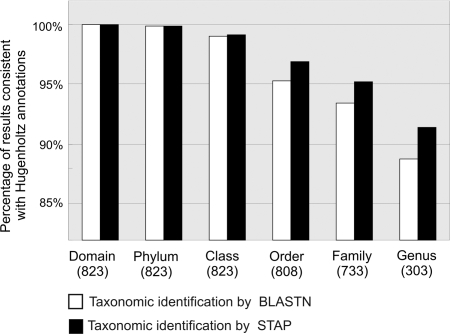
Comparison of reliability of BLASTN and STAP taxonomic assignments. The number below each taxonomic level indicates the number of bacterial sequences in the analysis that were annotated at that level (see Results and Discussion).

We compared the taxonomic assignments made by STAP and those made by BLASTN with the Hugenholtz annotation for the 823 bacterial ss-rRNA sequences from the complete STAP database which had more than six levels of taxonomic annotation. Based on this criterion, STAP outperforms BLASTN at all taxonomic levels (Figure 4). At higher taxonomic levels, there is little difference in reliability between the two methods. However, although domain-level assignments by BLASTN are as reliable as those by STAP, STAP also uses the domain assignment step to search for novel, deeply branching ss-rRNA sequences (see below) which would not be detected by BLASTN.

At lower taxonomic levels, the differences in reliability are greater. For example, of the 808 sequences with Hugenholtz annotations at the order level, STAP's assignments agreed with the Hugenholtz assignment for 783 sequences, while BLASTN matched in only 770 cases—i.e., a 1.6% difference between the STAP and BLASTN results at the order level. Similar comparisons demonstrated reliability differences of 1.8% at the family level and 2.6% at the genus level (Figure 4). At all levels, the STAP assignments were more consistent with the original ss-rRNA annotations than were those made by BLASTN. Further analysis of the phylogenetic trees confirmed STAP's greater accuracy in assigning taxonomic identifications at all levels (Table 2).

**Table 2:** Discrepancies between taxonomic assignments made by BLASTN and STAP.

Taxonomic Level	Phylum	Class	Order	Family	Genus
STAP more accurate	1	1	11	10	25
BLASTN more accurate	0	0	0	3	8
Unresolved	2	3	7	6	8

Bacterial sequences for which the assignments made by BLASTN differed from those made by STAP were identified, and the level of the Hugenholtz annotation for each was noted. Accuracy was scored based on comparisons with the Hugenholtz annotation. Those few cases where the BLASTN results matched the annotations but the STAP results did not were always found to be due to incorrect annotation in the Greengenes database for the sequence's closest neighbor in the tree. Sequences whose position in the STAP-generated tree was between neighboring groups were classified as “unresolved.”

Though the differences obtained here seem modest, it should be noted that because we divide the comparisons into six categories, the total difference is 10.3%. We believe that the differences are not only significant, but also underestimated since the criterion used was biased in favor of BLASTN. The comparisons were based on sequences with high-quality annotations which most frequently come from densely sampled taxonomic groups. BLASTN tends to perform well on such groups and to yield less accurate assignments for under-represented groups. As more sequences become available and are annotated, the under-represented groups will become better represented and thus BLASTN will come to perform better than it does currently. Nevertheless, there will likely always be an advantage to phylogenetic methods since these analyses can embody evolutionary processes as well as interpret the resultant sequences. Furthermore, whereas BLASTN simply compares the query sequence with a group of sequences, tree-based methods compare every sequence with all the others and take all the relationships into consideration.

The few cases where BLASTN gave more accurate results than STAP appear to be the result of inaccurate annotation of those sequences which happened to be the nearest-neighbor sequences in the STAP analysis. However, since STAP outputs the phylogenetic trees, erroneous assignments caused by individual database annotation errors can be easily captured and corrected. Such errors could be prevented by improved database annotation.

We have tested STAP against BLASTN for sequence datasets obtained from various environmental samples and found that the differences are highly sample-dependent: microbial ss-rRNA taxonomy assignments from human intestine reveal no difference between STAP and BLASTN, whereas deep-sea coral bacterial communities display a 4.8% difference [43], and sludge communities exhibit a difference of 8.3% [44]. Thus the advantage of STAP over BLASTN varies for different communities, and STAP has a clear edge over BLASTN in under-studied and complex communities because of its phylogenetic methodology.

Neither STAP nor BLASTN perform well when the query sequence is very distant from all known ss-rRNAs. In these cases, alignments will be poor and phylogenetic analyses may be prone to long-branch attraction. Potential solutions for these situations include the use of improved automated alignments based on secondary structure, manual curation of alignments for selected distantly-related sequences, and more adequate representation of all phylogenetic groups in the sequence databases.

### STAP produces alignments for massive sequence inputs

Many microbial ecologists need to align all the ss-rRNA gene sequences in one or more communities to each other for further analysis such as tree building for a whole community. Many currently available resources such as RDPII and Greengenes Project provide such service through their web servers [23], [24], while ARB software requires manual curation for alignment building (Table 1)[27]. And, notably, Greengenes' aligner NAST's alignment output is compatible for uploading into the ARB software, so it is commonly used for many ARB analyses [24], [27], [45]. A similar option is available in STAP via a profile-based ss-rRNA aligner.

We have tested the STAP aligner using a variety of data sets and the output is comparable to that produced by the Greengenes' web-based aligner and to alignments made manually in ARB. For example, we carried out a comparison of alignments produced for data from the human intestinal microbiota [28]. In the published study of this data an alignment was generated manually within ARB. We took the sequences from this study and ran them through the Greengenes aligner as well as STAP. To compare these three alignments, we selected 50 sequences representing the phylogenetic diversity of the intestinal microbial community, extracted the alignments of these 50 sequences from the larger alignments, and then used these alignments to generate phylogenetic trees (using a maximum likelihood method available on the CIPRES web-portal: http://8ball.sdsc.edu:8889/cipres-web). The resulting phylogenetic trees from each alignment were overall quite similar to each other although there are some small differences that suggest that the STAP alignment is as good and sometimes slightly better than the others. For example, to assess the accuracy of the trees based on the alignments, we compared the taxonomic assignments for each sequence with the structure of the phylogenetic tree. It is important to note that the taxonomy assignments are likely reasonably accurate as they are based on comparison to the entire ribosomal RNA databases while the trees are based on analyzing only the 50 subsampled sequences. Thus one might expect some taxonomic groups to be non monophyletic in the trees simply due to sampling artifacts. This is the case for both the manually aligned dataset (Figure S1) where sequences assigned to the Bacteroidetes branch within a clade of Proteobacterial sequences and the Greengenes' NAST alignment tree (Figure S2) where a sequence assigned to cyanobacteria groups within the Firmicutes clade. However, all taxonomic groups are monophyletic in the STAP alignment based tree (Figure S3), suggesting that a high quality alignment might make up for poor species sampling. Similar comparison of the STAP aligner and Greengenes's NAST aligner on other ss-rRNA datasets also indicates that overall these two programs produce comparable alignments.

Most microbial ecologists also want to know the identity (taxonomy) of the sequences in their ssRNA datasets. A researcher commonly has to switch back and forth between several sites and several methods to perform both tasks. The STAP ss-rRNA aligner and taxonomy assigner eliminates this inconvenience. STAP uses exactly the same phylogenetic approach to do both of these tasks (align and assign taxonomy) and integrates both into the same package. Both methods align the query sequence to a highly curated database and through phylogenetic iterations, produce either a gapped sequence alignment in fasta format (materials and methods) or the taxonomy associated with the query sequence's closest neighbor in the phylogenetic tree. The user can easily concatenate and merge both of these outputs for all of the sequences into one file. Notably, all the sequences will be aligned with each other because they have been aligned to sequences of the same length in the same database. STAP can take advantage of parallel linux computing to align individual sequences simultaneously, thus thousands of sequences can be aligned and assigned taxonomy in a matter of minutes, rather than the slow process involved with web servers.

### Identifying deeply branching ss-rRNA sequences

Although domain assignments by BLASTN are as accurate as those by STAP, using STAP's phylogenetic analysis for that step provides an added benefit—the ability to search for novel, deeply-branching ss-rRNAs. For this search, STAP builds a tree for the query sequence together with 340 representative sequences from the bacterial, archaeal, and eukaryotic domains. If the query sequence is found to branch near the node separating the three domains (as illustrated in Figure 2), there is a possibility that the query is from a novel, deeply-branching lineage and could be flagged for further investigation.

### Conclusions

We built STAP, a **S**mall Subunit rRNA **T**axonomy and **A**lignment **P**ipeline, as a tool to automatically generate and analyze high quality multiple sequence alignments and phylogenetic trees from massive amounts of ss-rRNA sequence data. It makes use of the publicly-available CLUSTALW, PHYML, and BLASTN packages, and automates the manual steps involved: gathering homologous sequences; building and masking multiple sequence alignments; and building and parsing phylogenetic trees. The pipeline is fast, robust and easy to implement, yet yields results for ss-rRNA sequences that are comparable to those achievable with manual phylogenetic analysis. Our comparative studies confirmed that tree-based methods are superior to approaches that rely on sequence similarity for inferring phylogenetic relationships.

STAP depends on the publicly-available ss-rRNA databases which are dominated by prokaryotic collections. The eukaryote functionality included in STAP provides structural completion and will need further development in the future. Likewise, STAP's accuracy depends heavily on alignment quality and annotation accuracy. An improved alignment algorithm combined with more accurate taxonomic annotation in the source databases is the key to improved STAP performance. Another future direction is to incorporate statistical analysis, such as the likelihood ratio test for taxonomy assignments [46].

### Availability

The STAP package includes the database and programs. Programs in the package include the scripts and modules described in the paper, as well as a profile based ss-rRNA alignment script to build alignments for large dataset. The STAP package is accessible from: http://bobcat.genomecenter.ucdavis.edu/STAP/download.html.

## Supporting Information

Figure S1 The maximum likelihood phylogenetic tree from the 50 representative human intestinal sequences aligned manually in ARB.
(8.30 MB TIF)

Figure S2 The maximum likelihood phylogenetic tree from the 50 representative human intestinal sequences aligned by Greengenes' NAST aligner.
(7.36 MB TIF)

Figure S3 The maximum likelihood phylogenetic tree from the 50 representative human intestinal sequences aligned by the STAP aligner.
(7.85 MB TIF)
