# Correction: An Automated Phylogenetic Tree-Based Small Subunit rRNA Taxonomy and Alignment Pipeline (STAP)

**DOI:** 10.1371/annotation/c1aa88dd-4360-4902-8599-4d7edca79817

**Published:** 2008-07-15

**Authors:** Dongying Wu, Amber Hartman, Naomi Ward, Jonathan A. Eisen

There was an error in the Funding section. The correct funding information is as follows: The development and main work on this project was supported by the National Science Foundation via an "Assembling the Tree of Life" grant (number 0228651) to JAE and NW. The final work on this project was funded by the Gordon and Betty Moore Foundation (through grants 0000951 and 0001660).

