# Supplementary figures and images for: An Automated Phylogenetic Tree-Based Small Subunit rRNA Taxonomy and Alignment Pipeline (STAP)
Source: PLoS One. 2008 Jul 2;3(7):e2566. doi: 10.1371/journal.pone.0002566 (PMC2432038; doi:10.1371/journal.pone.0002566)

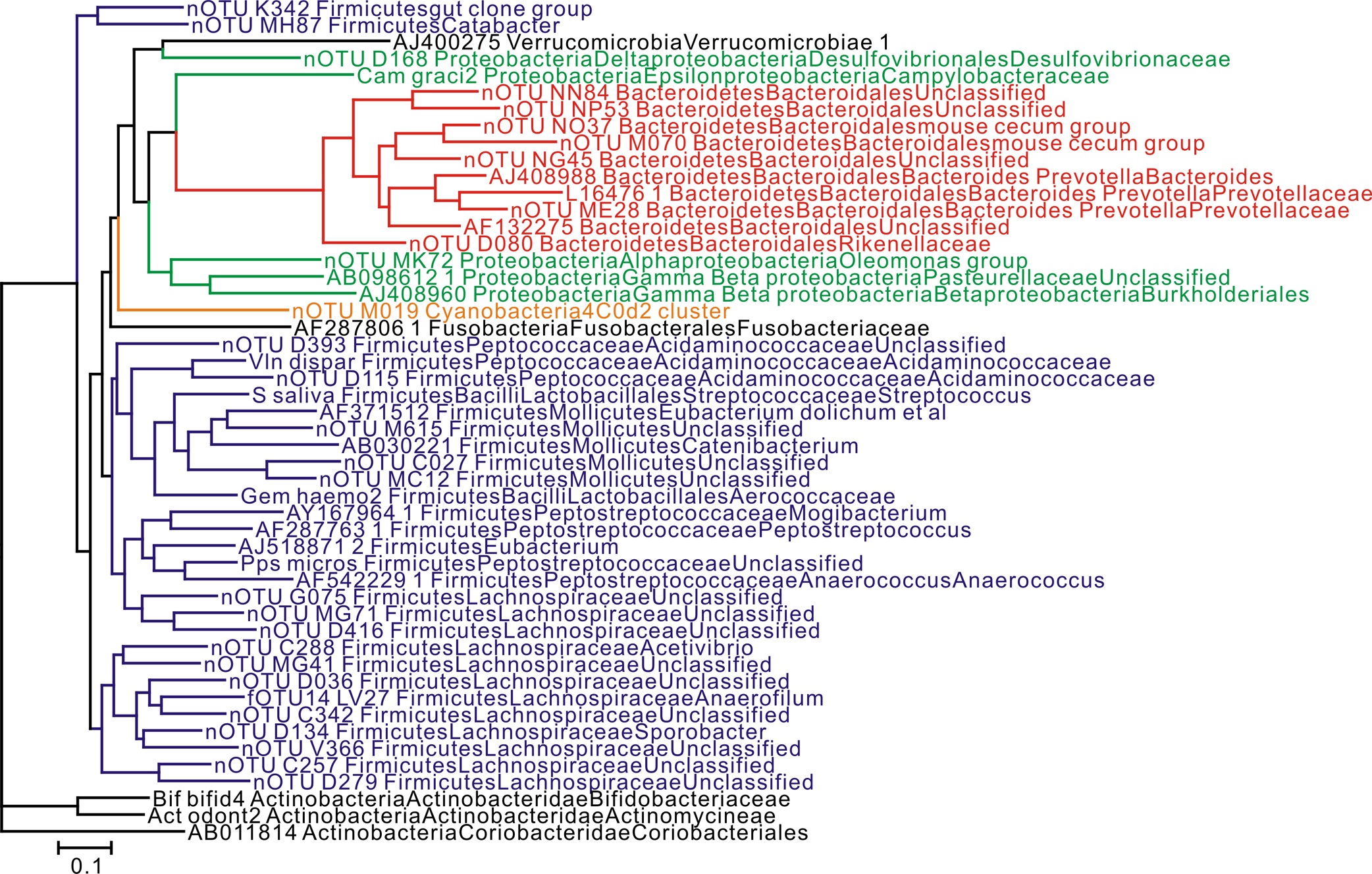

Supplement: Figure S1 — The maximum likelihood phylogenetic tree from the 50 representative human intestinal sequences aligned manually in ARB. (8.30 MB TIF) [file pone.0002566.s001.tif]

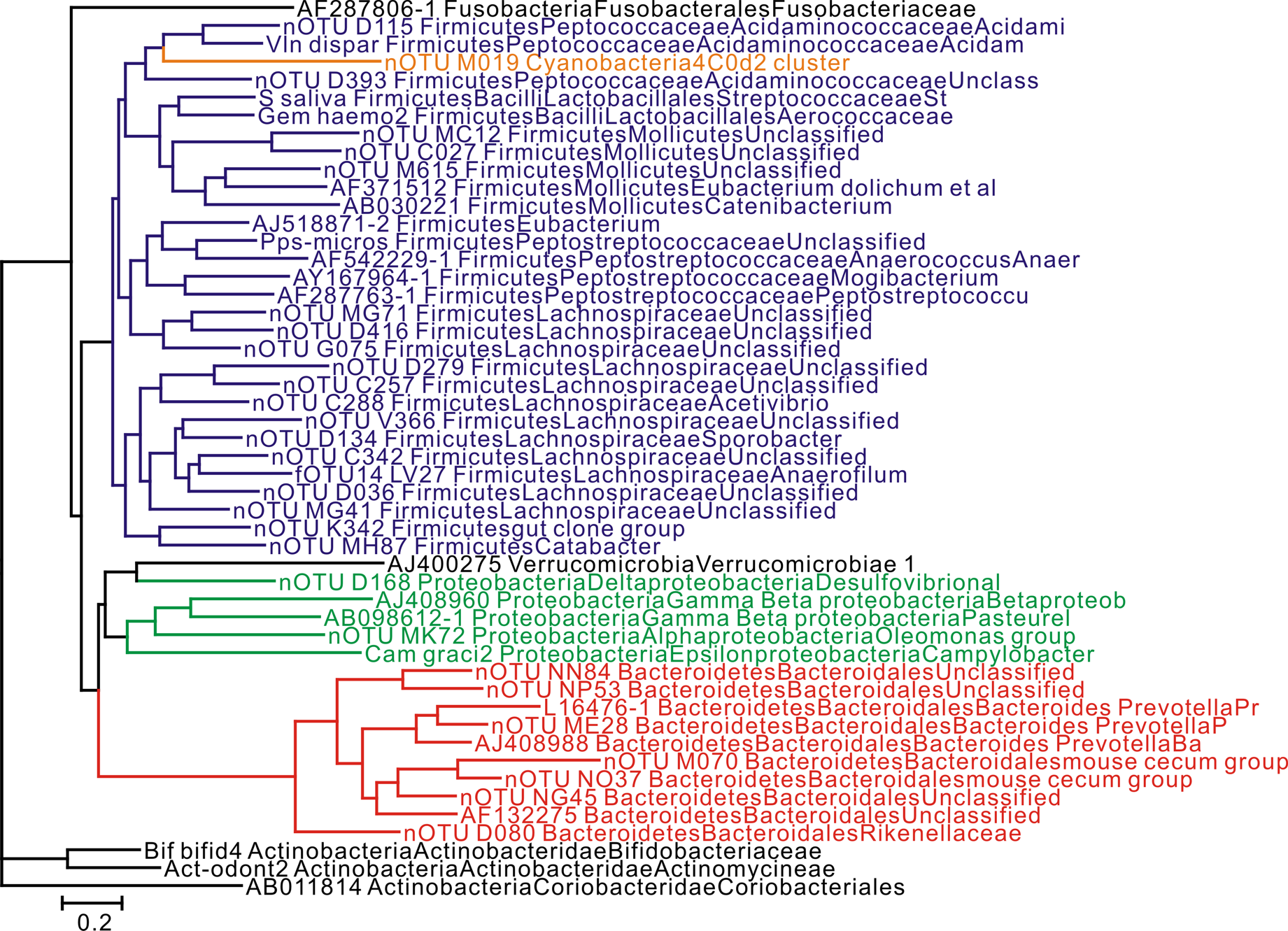

Supplement: Figure S2 — The maximum likelihood phylogenetic tree from the 50 representative human intestinal sequences aligned by Greengenes' NAST aligner. (7.36 MB TIF) [file pone.0002566.s002.tif]

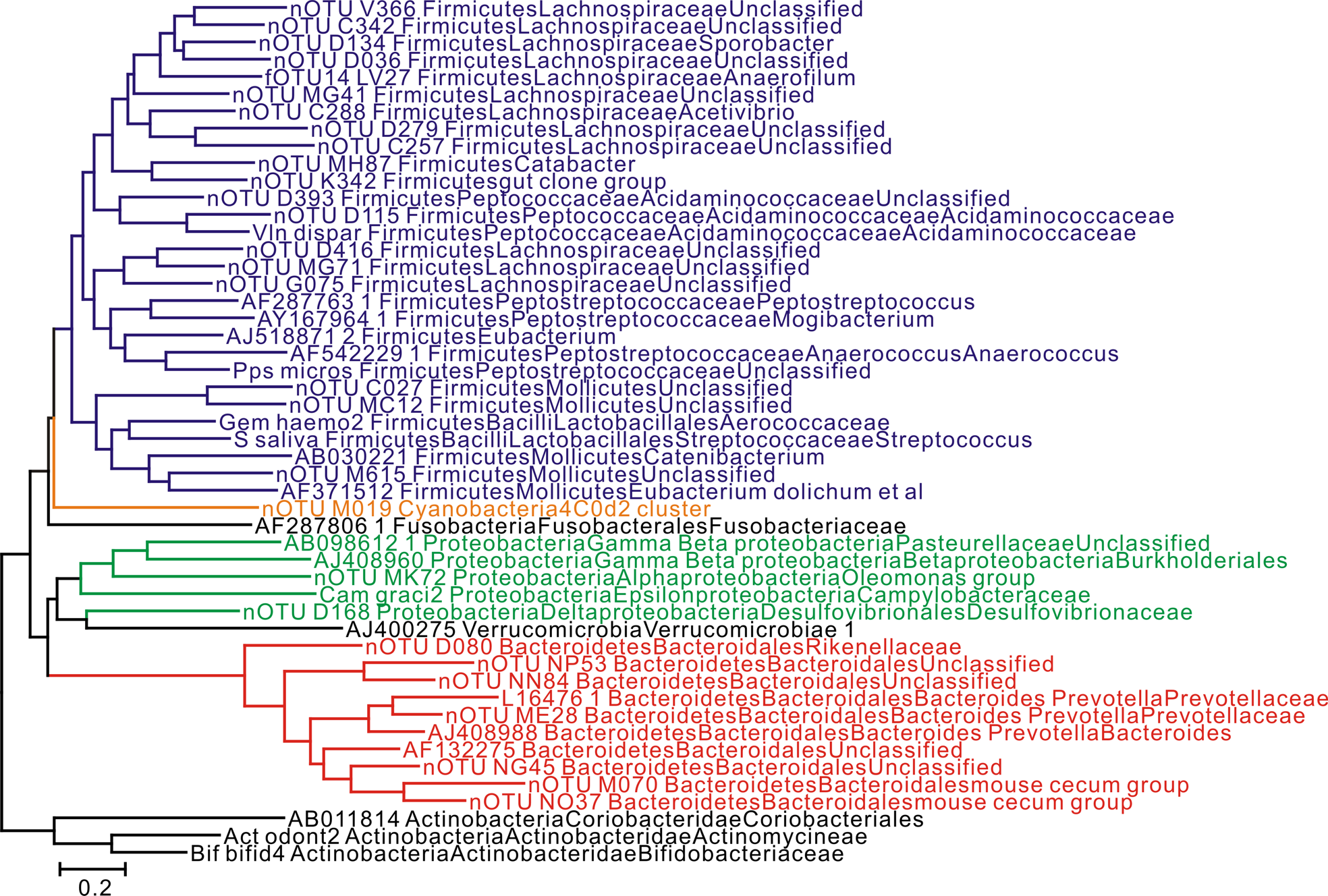

Supplement: Figure S3 — The maximum likelihood phylogenetic tree from the 50 representative human intestinal sequences aligned by the STAP aligner. (7.85 MB TIF) [file pone.0002566.s003.tif]
